# Resolutions of Respect to Dr. Jonathan Taft

**Published:** 1904-03

**Authors:** 


### Obituary.

### RESOLUTIONS OF RESPECT TO DR. JONATHAN TAFT.

Whereas, It has pleased the Divine Ruler to call into eternal rest Jonathan Taft, who passed the portals of the great unknown October 15, 1903, after a long and vigorous career of usefulness in the profession; and

Whereas, This Society especially feels his demise from the fact that one-half of the members constituting this body have received a large portion of their early dental knowledge and training directly from his lips; and we further recognize that he was unique in his power to impress upon the pupils who sat under his instruction sound principles of ethics and practice. He was great in his goodness, a characteristic which stands as a shining light for others to see and follow in his footsteps; therefore, be it

*Resolved,* That the Odontological Society of Chicago hereby testifies to the loss experienced by the profession in the death of Dr. Taft, and extends sympathy to the family in their bereavement; also

*Resolved,* That these resolutions be spread upon the records of this Society, and that a copy be forwarded to the dental journals for publication.

(Signed) J. G. Reid,

L. L. Davis,

J. W. Wassall.

Adopted January 12, 1904.

